# Qualitative Examination of Cooperative-Intelligent Transportation Systems in Cities to Facilitate Large-Scale Future Deployment

**DOI:** 10.3390/s22218423

**Published:** 2022-11-02

**Authors:** Shuo Li, Simon Edwards, Memduh Ozer Isik, Yanghanzi Zhang, Philip T. Blythe

**Affiliations:** School of Engineering, Newcastle University, Cassie Building, Claremont Road, Newcastle upon Tyne NE1 7RU, UK

**Keywords:** cooperative intelligent transport system (C-ITS), cooperative vehicle–infrastructure systems, qualitative research, semi-structured interviews, thematic analysis, stakeholder analysis

## Abstract

The rate of urbanization in Europe is increasing rapidly. Traffic congestion has become one of the biggest challenges for cities. Additionally, thousands of people die each year in accidents on European roads. In addition, road transport is one of the biggest reasons for the increase in air pollution and greenhouse gases in Europe. To solve these problems, cooperative intelligent transport systems (C-ITS) have accelerated in Europe, after more than ten years of research and development. The European Commission has carried out significant work in this field in recent years and has prepared a strategy document for the deployment of C-ITS services in Europe. The Commission considers that C-ITS have significant potential in reducing the negative effects of road traffic and expects these systems to deploy rapidly in European cities. However, in order to achieve this, it is imperative to clearly identify the needs of cities in implementing and managing these systems, the extent to which these systems will respond to different mobility problems of the cities, and the important barriers to widespread deployment. This study focused on qualitatively examining the C-ITS deployment from the stakeholder perspective. The knowledge generated is useful to facilitate the large-scale future deployment of C-ITS.

## 1. Introduction

Urbanization is a global phenomenon. The latest report published by the United Nations [1] indicates that urban population will increase from 56% to 69% by 2050. According to the same report, European urbanization rates are much higher, constituting 73% of the total population, and predicted to increase to 86% by 2050. This trend of urbanization will lead to a significant increase in the number of vehicles on urban roads.

The European Commission (EC) regards the development and deployment of cooperative intelligent transportation systems (C-ITS, also known as cooperative vehicle–infrastructure systems) as crucial to counteracting the adverse safety, efficiency, and environmental impacts of increasing road transport. It supports the potential for developments in telecommunication technologies to be applied to transport, whilst also engaging with stakeholders to maximise the benefits of C-ITS, including enhancing road safety, reducing traffic congestion, better management of the existing transportation infrastructure, increased mobility, better travel time reliability, and increased efficiency of passenger and freight transport [2]. A range of specific C-ITS services were identified initially by the C-ITS deployment platform (2014 onwards) and further defined by the Amsterdam Group (a strategic alliance of stakeholders facilitating deployment of C-ITS). They focused on:

- Efficiency (green priority; parking; flexible infrastructure (e.g., peak hour lanes); in vehicle signage; mode/trip time advice).
- Safety (road hazard warning; red light violation warning; pedestrian warning; P2W/cycle detection; blind spot detection; emergency vehicle warning; road work warning).
- Environment (green light optimal speed advisory—GLOSA; eco-driving; speed advice).

C-ITS rely on smart components on both vehicles and the roadside infrastructure, with suitable communication technologies essential. The EC is committed to establishing a standard for C-ITS services to communicate securely with each other, based on two means of communication:

ITS-G5 (IEEE 802.11 or Wireless LAN): when two or more vehicles or stations are in radio communication range, they connect automatically and establish an ad hoc network; the range of a single link is only a few hundred meters, so every vehicle becomes a router, which enables multi-hop messages to communicate with other vehicles and stations.

Cellular-based: this approach is becoming more widespread for already existing G5-based short-range communications, especially vehicle-to-everything—V2X—services; it utilises existing cellular networks for long-range communication; cellular technologies can connect vehicles to infrastructure via cloud services and backend interfaces (vehicle-to-network).

It is likely that future C-ITS deployments will be hybrid communication approaches based mainly around cellular communications, with additional bespoke G5 services.

Stakeholder engagement is also an important component for strategies to roll out C-ITS services. The C-Roads Platform was set up for authorities and road operators to harmonise roadside C-ITS deployment across Europe; it links all ongoing C-ITS deployments [3]. The CAR 2 CAR Communication Consortium (C2C-CC) is an automotive industry driven organisation (vehicle manufacturers, equipment suppliers, research organisations, and other partners) [4]. It is imperative to understand and evaluate the roles, responsibilities, and needs of stakeholders.

It is clear that there are many factors to be considered for the development and large-scale deployment of C-ITS. The enrichment of in-vehicles technologies, infrastructure development, standardisation, regulation, and security are vital factors [5]. C-ITS is built on existing ITS technologies and research, for ITS is the basis for C-ITS. This means existing transport equipment, technology, and transportation centres are also used for C-ITS, as well as the concepts of ITS, such as data collection, analysis, and communication architecture. All these create the foundation for C-ITS [6]. The difference between ITS and C-ITS is that, while ITS provide digital tools to create intelligence and data on roadside units or in vehicles, C-ITS provide two-way communication between vehicle-to-vehicle (V2V), vehicle-to-infrastructure (V2I), vehicle-to-pedestrian (V2P), vehicle-to-network (V2N), and vehicle-to-everything (V2X).

Whilst C-ITS can be considered a self-sufficient concept, based on ITS with bespoke deployments, it is also extremely important to understand that it is a component in other concepts, such as smart cities. Moreover, it is a crucial ‘stepping-stone’ towards connected and autonomous vehicles (CAV). In this case, it is not only the technology development that is important, but also stakeholder engagement and deriving learnings to inform policies. Indeed, the EC’s aim is to create a reference framework, in order to provide cooperative, connected, and automated mobility (CCAM) policies.

**Figure 1:**
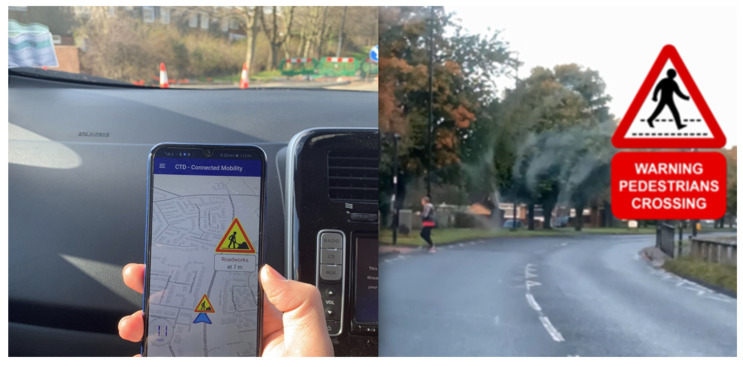
Examples of C-MobILE C-ITS services—the RWW system (**left**, Road Work Warning—informs drivers in a timely manner about road works, restrictions, and instructions) and the concept WSP system (**right**, Warning System for Pedestrians, detects risky situations, e.g., road crossing, involving pedestrians, allowing the possibility to warn vehicle drivers).

The Horizon 2020 Research and Innovation Programme has also been key to promoting and investing in C-ITS. The C-Mobile project (accelerating C-ITS mobility innovation and deployment in Europe) deployed C-ITS services in eight cities (Barcelona, Bilbao, Bordeaux, Copenhagen, North Brabant, Newcastle, Thessaloniki, and Vigo) [7]. The project ‘integrates C-ITS concepts in practical, real-life and complex environments that aims to provide safe and efficient road transport without casualties and serious injuries on European roads, in particular in complex urban areas and for vulnerable road users’. A wide range of C-ITS services were deployed in the C-MobILE project [8]. These services are grouped into four categories, according to their functionalities—urban efficiency, infrastructure-to-vehicle safety, traffic efficiency, and vehicle-to-vehicle safety. The example of C-MoBILE C-ITS services area is illustrated in Figure 1.

### 1.1. State of the Art and Research Gaps

As road transport increases, it can result in inefficiency, as well as environmental and safety disbenefits. Private car ownership has been increasing in Europe, and it is possible that this trend will continue in the future, based on urbanization trends, and may even accelerate because of COVID-19, which hit mass public transport hard. These trends have pushed ITS to the forefront of transport policy in the hope that it could alleviate the problems of road transport congestion worldwide. C-ITS, an intelligent transportation approach that can respond to the transportation trends of the future, in line with developing technologies and constantly changing transportation needs, has emerged, and its importance is increasing. Academics, industry, and governments are considering C-ITS as a crucial component in packages of solutions. The European Commission has ensured that C-ITS services are designed to benefit road transport, along with wider safety, efficiency, and environmental concerns, and fully support deployment activities. Existing research about C-ITS technologies mainly focus on the evaluation and simulation of the performance and effectiveness of C-ITS systems from the road efficiency and environmental and safety perspectives. Eckhoff et al. [9] found that the C-ITS application-Green Optimal Speed Advisory is capable of cutting CO_2_ emission and fuel consumption by about 13. Edwards et al. [10] evaluated serval C-ITS systems (red light violation warning, road hazard warning, energy efficient intersection, green light optimal speed advisory, time-to-green, and green priority) using both field trails and microscopic simulation. They found that C-ITS systems resulted in a 2–6% efficiency savings for both light and heavy vehicles and a cut of over 200 g CO_2_ per bus route per trip for one site. Hajiebrahimi and Iranmanesh [11] proposed and simulated the performance of a C-ITS application—Smart Traffic Control (STC)—and found that it resulted in a 12% cut of delay for emergency vehicles and a 18% cut in delay for ordinary vehicles, compared with existing well-known traffic control methods. Additionally, Jan et al. [12] simulated a C-ITS application—optimal restricted driving zone (ORDZ)—aiming to choosing suitable restricted traffic zones to reducing road congestions and traffic pollution, and the results showed that ORDZ performed better than existing methods and received the most improved satisfaction among citizens.

The potential benefits of C-ITS system, in terms of contributing to improved road efficiency, air quality, and road safety, has been recognised by existing studies. However, knowledge for understanding the deployment of the C-ITS services from the perspective of the key stakeholders who have been actively involved in the deployment process is significantly unresearched. Limited focus has been paid on identifying the key issues and barriers of the large-scale deployment of the C-ITS services. In this context, the research presented in this paper provides a political and technical background for C-ITS. Then, it represents experts’ opinions about the deployment process. These insights are conveyed in a thematic approach and provide information on the benefits of C-ITS services for cities, based on real experiences, the additional needs of cities, and barriers to widespread future expansion. Therefore, the paper provides important information about C-ITS deployments and provides an advanced level of understanding providing a more robust academic evidence base to complement existing stakeholder engagement activities. This study could, therefore, assist strategy formulation for cities across the world that want to deploy C-ITS services in the future.

### 1.2. Purpose of the Research

To fill the research gaps above, the overall aim of this study is to advance an understanding of C-ITS deployment and identify the key issues and barriers for a large-scale deployment, within the context of the European guidelines for the deployment of C-ITS services, particularly in an urban setting.

## 2. Materials and Methods

### 2.1. Participants

The participants were recruited through a mailing list of key C-ITS stakeholders, including policymakers, service providers, and academics. In total, four experts participated in this study. They were from Newcastle, upon Tyne and Birmingham, both UK. The detailed descriptions of their backgrounds and expertise are summarised in Table 1.

### 2.2. Research Design and Data Collection

The aim of this study is to broaden and deepen the understanding of C-ITS deployments and identify the key issues for a large-scale deployment, within the context of the European guidelines for the deployment of C-ITS services, particularly in an urban setting. The nature of this study is qualitative. It is not aimed at generalization, but is focused on generating a rich and contextualised understanding of human experiences [13]. In qualitative studies, focus groups and interviews are the most widely used data collection methods [14,15,16,17]. Compared to focus groups, interviews were more suitable for this study. The reason for this is that each participant has extensive experience in the field of C-ITS, so it is important to provide them with sufficient time to share their perceptions, experiences, and opinions fully [14,17]. Due to the COVİD-19 pandemic, all interviews had to be conducted online. The interviews were semi-structured, and the structured topics covered the key issues regarding the deployment of C-ITS; they were derived from previous literature, as follows:

- C-ITS applications deployed and general opinion towards them.
- Key questions/issues prior to commenting, regarding continuation and future expansion of the C-ITS services.
- Cost for implementing C-ITS applications.
- Live costs of C-ITS (maintenance, repair, upgrade, and replacement of system elements).
- Involvement of multiple partners.
- Barriers and challenges in the deployment of C-ITS.
- Recommendations and advice for future deployment of C-ITS in other cities.

### 2.3. Data Analysis

**Figure 2:**
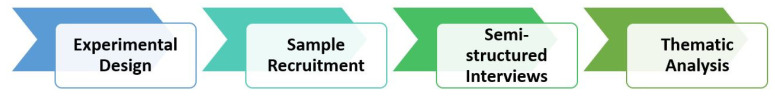
Research process flowchart.

The research process is illustrated in Figure 2. The collected qualitative data was analysed using thematic analysis, which is a method for understanding, identifying, analysing, and reporting patterns within qualitative data [18]. The thematic analysis consists of six key stages, as defined by Braun and Clarke [19], including data familiarization, initial coding, searching for themes, reviewing themes, defining themes, and reporting. The thematic analysis was executed using NVivo, which provided a clear environment to analyse data and enabled the process to be more effective and efficient. NVivo 12 is a qualitative analysis programme. NVivo provides a great environment to organise, store, and analyse data. It provides more efficient data analysis process and permits creation of visual maps to examine raw data easier.

## 3. Results and Discussion

### 3.1. Data Familiarisation

**Figure 3:**
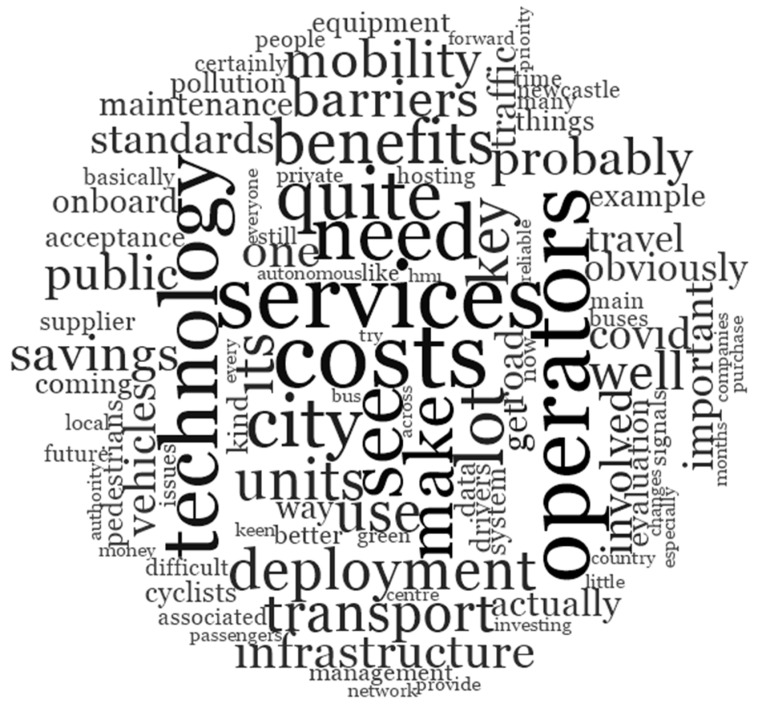
Word Cloud in NVivo 12.

The first step of thematic analysis is data familiarization, as suggested by Braun and Clarke [19]. A word cloud figure as generated to virtualise the data. As Figure 3 shows, some words are particularly prominent. The words “costs”, “operators”, “services”, “technology”, and “needs” are prominent words, some words, such as “infrastructure”, “COVID”, and “barriers”, are also among the words that are mentioned in significant amounts. Table 2 also shows the weights of the most frequently mentioned words in the data. 

### 3.2. Coding

**Figure 4:**
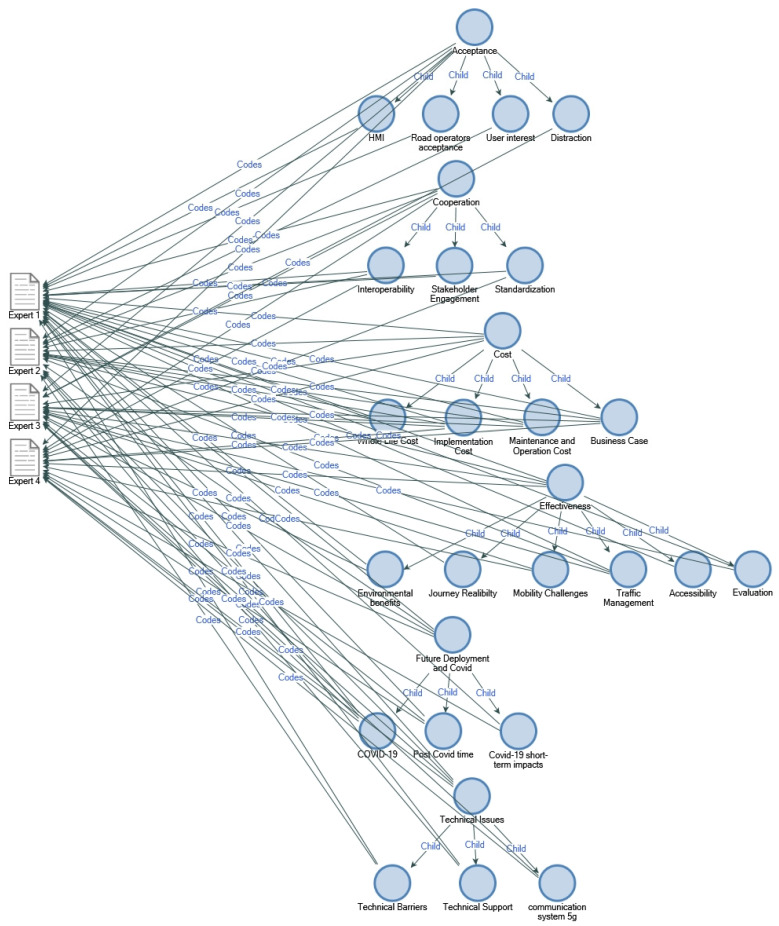
Thematic analysis coding map.

The thematic analysis initially resulted in 84 codes, and the 84 codes were grouped in to 23 sub-themes. All themes were re-evaluated to make the research more effective and understandable. The significance of the data was taken into account, and finally, the 23 sub-themes contributed into 6 core themes. The thematic analysis is presented in Figure 4, which illustrates the linkage between the participants and the key themes identified. Additionally, the core themes are summarised in Table 3.

#### 3.2.1. Theme 1. Cost

The first theme is cost, which is key for the deployment of C-ITS. Experts made price assessments of C-ITS of different dimensions. Participants commented on issues such as providing appropriate infrastructure and communication systems and, later on, the operating costs of C-ITS systems. Thus, the opinions of the experts created three sub-themes under the theme of cost. These are implementation costs, operation and maintenance costs, and whole life costs.

- Implementation Costs

Participants’ views on C-ITS implementation costs were generally related to the provision of appropriate communication infrastructure and equipment prices, such as ITS-G5 technologies and onboard units. C-ITS implementation costs are expensive, and E1 provided the following comment, in relation to implementation cost:

‘*I think for the ITS-G5 technology, which is the technology that’s being deployed on the buses, and that requires quite a lot of hardware in terms of roadside units, usually located at traffic signals or pedestrian crossings. And also, onboard units on the vehicles, which would usually consist of an onboard unit and an HMI. So, there’s quite a lot of capital investment for equipment. And then there’s the whole software side of things as well and hosting servers, sending things to the cloud, and making sure the HMI is user friendly and developed in that sort of way. So, there is quite a lot of money involved*’

(E1)

The participants believed that the implementation costs could be reduced with the development of technology applications. Example quotes from E1 and E2 are as follows:

‘*I think the future is C-ITS services delivered through cellular devices and to smartphones using apps. I think it’s probably more user friendly and probably cheaper in the long run than the sort of ITS-G5 system*’

(E1)

‘*Each traffic signal needs to have a roadside unit probably about 5000 pound worth of equipment. And each vehicle needs an onboard unit which is at the moment running at about 1500 pound. Now from this year, all new XXX will come equipped with the technology to communicate, so these costs will become cheaper as time goes on*’

(E2)

Another point participants raised, regarding the implementation cost, is the cities’ existing infrastructure. The participants mentioned that, if a city has a modest communication infrastructure or SCOOT system and if they use their existing infrastructure effectively, the deployment of the C-ITS services could be relatively easy, without extra investments. Example quotes from participants are as follows:

‘*You can deliver a system that takes advantage of some of the infrastructure that you may well already have, if you’ve got a SCOOT system*’

(E3)

‘*I think, the comms and some of the other hardware aspects don’t have to be expensive, but we might need to think about creative solutions*’

(E3)

- Operation and Maintenance Costs

The participants commented that the operation and maintenance costs are cheaper than implementation. Example quotes from participants are as follows:

‘*When you think you’ve got, you know, 20 signals in a test area, or 200 signals across the city, then that’s a lot of money. 200,000 pounds for small area 2 million for your entire city, just to put the new stuff in. And then if you’ve got, say maybe maintenance costs of a few hundred to a few thousand per signal each year, you know, you’ve got these ongoing costs maybe 20 30 40 50,000, or whatever, but it’s still going to be a small but not insignificant percentage of what you’ve already spent*’

(E4)

‘*I don’t really imagine too much in the way of maintenance being required, because it will be part of the wider refresh of your problems and your CPUs inside your traffic systems, etc.*’

(E3)

Participants believed that it would be costly to keep the system up to date, but fortunately, the technical support, such as support for road users or training staff to use the system required for the continuity of the system, is relatively less expensive. Example quotes are as follows:

‘*There’s maintenance and operational costs going forward. Potentially upgrading standards, which is quite significant. So, the latest standards are 2019. But as I say the deployment in XXX is still 2015. And they’re looking into upgrading to 2019. And there’s costs associated with that, as well*’

(E1)

‘*The cost of training users and staff are relatively low*’

(E2)

- Whole Life Cost Analysis

The participants stated that the whole life cost is not clear for their projects. The cost benefits analysis for C-ITS applications is a common issue for European countries. In terms of the UK, the webTAG is not suitable for assessing C-ITS applications, as the C-ITS projects are relatively small. Another important point is that there is a business case barrier, as the effectiveness of C-ITS applications is not as clear as expected. Because of this, it could be difficult to build a strong business case and to obtain suitable funds for C-ITS projects. Example quotes are as follows:

‘*There’s a business case barrier because we need to see the measurable benefits from this technology to make it worth investing in*’

(E1)

‘*They also have to work reliably and all the time. This probably needs a paragraph at least in the conclusion, if there isn’t one already. It’s actually crucial else drivers become frustrated and lose confidence in the system*’

(E1)

‘*CITS applications are generally quite small, I mean, you know, in (a previous EC project) compass4D, we’re talking about a small area of the city with 21 junctions and C-ITS corridors there’s something like 18 junctions, and there’s maybe 30 buses involved. So, while we can show that there’s a benefit to those vehicles, there’s not necessarily a clear and tangible benefit to the whole of the network or the whole of society, or everybody using the road. And because it’s such a small area.*’

(E4)

The first key theme identified via the thematic analysis is cost, which is a key factor for C-ITS deployment. The estimated cost for inefficiencies in urban mobility and road congestion is 110 billion Euro per year, which is more than one percent of gross domestic products (GDP) of the EU [20]. Additionally, road transport is recognised to be responsible for a great amount of transport emissions, including greenhouse gases and air pollution [2]. The expected outcome of a C-ITS deployment is to not only gain cost benefits by enhancing road efficiency and reducing congestion, but also to provide a clean and safe road transportation system [2]. The findings of this study showed that the implementation costs of C-ITS are expensive, due to immature technologies. Although maintenance and operation costs are relatively less expensive, there is an important business case barrier. This prevents rapid deployment and reduces the expected benefits. This finding is in accordance with the fact that European Union [2] expected the deployment of C-ITS day 1.0 services (the first tranche of services to be deployed) between 2018 and 2030 to provide a 3 to 1 benefit cost ratio, and the high overall benefits depend on rapid deployment. Therefore, slow initial uptake in long periods could lead to less benefits. In addition, as part of the C-ITS deployment in C-Mobile, Mistakis and Kotsi [8] conducted a price benefit analysis for eight European cities. The findings were obtained based on C-ITS services impact data, C-ITS components costs data, and deployment sites data (i.e., the current status of C-ITS infrastructure and end users).

According to findings by Mistakis and Kotsi [8], the cost benefit analysis that results from the spread of C-ITS day 1.0 services in five out of eight cities is below the expectations of the European Union strategy. The BCR result for Bordeaux is 0.5, which could be because of the city’s deployment plan for all twenty C-ITS services across most of the city network [21]. This corresponds to the findings of this research that, in order to reduce the cost of C-ITS deployment, it is important to develop a realistic deployment strategy corresponding to a city’s current situation. Additionally, the experts suggested that, thanks to new technologies, the cost of C-ITS could be decreased. In the future, with the fast development in communication technologies, as well as the more mature C-ITS services, the implementation of the C-ITS services could be reduced, and the expected benefits could increase.

#### 3.2.2. Theme 2. Acceptance

The second theme identified is acceptance. The participants generally believed that the C-ITS services deployed are easy for the end-users to adopt. In addition, the participants believed that, in order to further deploy C-ITS services on a large scale, the acceptance from all stakeholders, including all motorised road users, is important. Example quotes are as follows:

‘*I think we have to have the acceptance of the fleet operators. So, engagement with the stakeholders is really important*’

(E1)

‘*The users have been keen to be involved. They are interested in any technologies that improve savings for the operator, potentially improve safety and make the driver’s life easier. And the drivers themselves are quite keen. I think they’ve shown quite a lot of enthusiasm for these technologies*’

(E1)

The findings highlight the importance of the acceptance of C-ITS for a large-scale deployment. The findings correspond with [2], that it is important to ensure the deployed C-ITS services meet the expectations of all stakeholders, not only individual car drivers, but also professional drivers, fleet owners, and infrastructure owners.

The participants pointed out that the design of the human–machine interfaces of the C-ITS service is the key to end-users’ acceptance and adoption. They raised a concern that, even if there is positive expectation, in terms of individual drivers’ adoption and acceptance towards C-ITS services, less user-friendly design of the human–machine interfaces may result in these services becoming distractions for drivers and then reduce end-users’ acceptance. Additionally, a good design and planning process of C-ITS projects is important, in order to provide effective use of technologies. When preparing projects, environmental factors, geographical conditions, existing infrastructures, and services should be evaluated well. In-vehicle C-ITS technologies should be designed to be as advanced as possible, but not overload the driver with excessive information.

Example quotes are as follows:

‘*When we use the ambulance service for that they’d be driving hundreds of miles a day. But for most of the day, they weren’t in areas with technology that were equipped. They said, six out of seven hours, I saw nothing on the app. So, when they did get to the area where the app was doing something, they’ve lost interest in it, because it’s been so long, just displaying the logo*’

(E2)

‘*We would not design the user interface for how you would deliver C-ITS in the car. That would be with the manufacturers. From my perspective, I definitely think you know, it’s another distraction. A GLOSA is another distraction for the driver. That may or may not offer some benefit. There was already evidence of people in the trial saying, Yeah, yeah, if I focus on this, I can just ignore the traffic lights and these kinds of comments*’

(E3)

‘*When you’re within 50 m of a junction, simply you should not be looking at a screen, you should be looking out and around the junction. So, I think there were safety issues with the design of both the interface for CITS Systems and how they display information and feedback to the user in a clear and concise manner. Now if the system’s asking you to do something complicated, how do you display that to the driver without overloading them with information?*’

(E4)

‘*I would suggest that the design of the HMI is really important*’

(E1)

#### 3.2.3. Theme 3. Effectiveness

The deployment of C-ITS services is intended to improve road safety, air quality, and travel reliability. The participants believed that the effectiveness of C-ITS for mobility challenges and traffic management is an important factor for deploying C-ITS services in cities in the future. Example quotes are as follows:

‘*We are aware that air pollution, for example, is a massive issue at the moment. So, C-ITS in XXX, it’s kind of a little bit experimental still. We’ve had projects going back to 2013 in C-ITS. And the main services have been services that help save energy and reduce pollution and save costs for fleet operators*’

(E1)

‘*It might be possible to deliver, you know, maybe a 10% journey time saving through the junction through the area of the junction. But, obviously, GLOSA doesn’t work during the peak hour, which is again, where our efforts would be focused. And so, it’s not really going to deliver on our congestion objectives*’

(E3)

‘*A lot of these technologies seem to be relatively limited. At the moment, a lot of the focus of the work that we do is on traditional transport planning and traditional kind of road building and cycle tracks and all that kind of stuff*’

(E3)

The above quotes show that experts think that C-ITS services show the potential to reduce air pollution, encourage energy efficiency, and enhance road safety. However, the participants believed that the current C-ITS technologies are not enough to fully deliver cities safety or environmental related objectives. Additionally, experts’ opinions have revealed that evaluation is a key issue, especially for local authorities.

‘*Evaluation is quite a tricky one*’

(E1)

‘*We were probably on quite a low TRL level in terms of where the research was. So, we were never looking at a solution in a scientific way. We look at it as a local authority that was basically saying, can we get the system to work?*’

(E3)

In terms of traffic management, experts believe that C-ITS services have the potential to deliver benefits to the city’s traffic management.

‘*It offers us opportunities to be able to better manage traffic that’s on the network. Because more automation more data and what have you give us, you know, a better overview and allow us to fulfil the aims of the Traffic Management Act*’

(E3)

‘*If you provide more reliable journeys, passengers are more likely to use that mode of transport. And so that would help the city towards their overall transport objectives, which are to make public transport more attractive than private vehicles for people to use when they commute or travel for leisure. And from a management point of view, obviously the management centre controls the traffic signals so they can obtain various network benefits*’

(E1)

Cooperative ITS for Mobility in European Cities [21] conducted a study about the urban benefit of C-ITS. The result shows that C-ITS is expected to provide a wide range of benefits for both road safety and transport efficiency. The safety impact of C-ITS is slightly high, when compared to benefits or expected benefits of ITS and connected driving. This is in accordance with the findings of this study, which also showed that C-ITS services are useful for increasing road safety and reducing the air pollution and greenhouse gases coming from road transport. Additionally, participants stated that advanced C-ITS technologies could potentially provide great benefits for traffic management in cities. One important issue, related to the effectiveness of the C-ITS services, is that some services, such as GLOSA and green priority, do not work at peak times, and participants thought that C-ITS technologies are not mature enough to fully deliver their expected benefits. Additionally, evaluation is an important issue for local authorities. Although their estimation is that GLOSA provided 10% journey time savings, they accepted that, rather than a scientific evaluation, they are trying to keep the system working properly. Without a clear and effective evaluation methodology, the safety and environmental benefits of C-ITS services could not be fully shown, which leads to poor business cases.

#### 3.2.4. Theme 4. Cooperation

C-ITS is a complex system. Participants mentioned that the cooperation and collaboration between different sectors and partners are important for the deployment of the C-ITS services. They included policymakers, technical partners (hardware and software), and the individual drivers. Moreover, participants also mentioned interoperability and standardization. The deployment of these systems is tightly linked to these two points. Therefore, two key sub-themes, partnership and interoperability, have been created in this theme.

- Partnership

In order to deploy C-ITS services, collaboration between partners is needed. Participants mentioned some important requirements for a good partnership. The most remarkable points are good communication between partners, good knowledge and related skills about services, and compatibility between services providers (using the same standards).

‘*We worked with a company called XXX for the onboard units, and we worked with a company called XXX for the HMI. And obviously, we’ve worked with operators. I think that the answer is good communication and clear instructions from the city as to what they want*’

(E1)

‘*The biggest problem we’ve had is that we all must work to the same standards. And those standards are interpreted, by different equipment providers, in different ways*’

(E2)

‘*The biggest problem is more related to skills. And you need somebody who’s got a good knowledge of traditional IT stuff—what is an API and how the systems work—paired with people who also strongly understand the transport system, and there aren’t that many of those and I think that was the biggest problem is finding people with enough expertise …… So, they’re able to talk about C-ITS as a concept and standards etc. But when it comes down to having the actual skills and the understanding to design and implement these systems, I felt that was quite lacking in terms of them*’

(E3)

- Standardization

Participants also indicated that standardization at the European level is a key facilitator for deployment. However, the participants mentioned that this will not be easy because of geographical issues and city-specific contexts, but it is important to research towards optimising standardization. Example quotes are as follows:

‘*We need to talk to other cities who’ve deployed C-ITS and understand if they’re doing something slightly differently and getting more benefits or getting less benefits. So, it’s really important that we all network with other cities*’

(E1)

‘*I think the interoperability is the whole point of international standards. If I take something from XXX, it should work in XXX. There might be certain differences in terms of geography, that could be a problem. Maybe you’ve tested your CITS system in a very flat area, with really good network coverage with a bunch of low buildings around so there’s not many multipath signals, bouncing off everything and the system works really well. Whereas if you took it to a mountainous region, and you’ve got a city with maybe tall buildings and steep street canyons, you know, worst case scenario. Then suddenly you find that you can’t get radio signal, or your GPS drops out all the time. And the position of the vehicle isn’t known*’

(E4)

‘*I think I would also support the standardization process making sure that if you use icons, for example, on the HMI, they’re all the same across Europe. So that you have that kind of common understanding of what something means*’

(E1)

The findings of the research showed that cooperation is quite important for the large-scale deployment of C-ITS. The cooperation theme created two important key sub-themes: standardization and partnership. The findings of this study correspond to one of the six key components of the European C-ITS strategy—international cooperation [2]. According to the strategy, international cooperation is fundamental because C-ITS technologies are new and when public authorities deploy services, they learn new things from each other [2]. Additionally, the strategy mentions that the EU has benefitted from cooperation with different countries. The EU wants to continue this cooperation and provide a common international standardization for C-ITS. The current findings showed that, if an international standardization is not provided, C-ITS deployment in a large-scale could not be possible because different suppliers use different standards, and this situation leads to many problems in the implementation, maintenance, and operation processes of C-ITS services. Apart from these, C-ITS is complex and needs many stakeholders for implementation and operation. The findings showed that good communication and good knowledge among stakeholders are important. These can be provided by a good business case, standardization, and good understanding of the European C-ITS deployment framework and technologies adopted within the framework.

#### 3.2.5. Theme 5. Technical Issues

In previous themes, the participants have mentioned several points relating to the deployment of C-ITS services, including the cost of communication systems, as well as user acceptance of C-ITS services. This theme focuses on issues mostly caused by problems on the technical side. One important technical barrier is finding a supplier. Some C-ITS services and technologies are immature. Example quotes are as follows:

‘*There are actually technical barriers. Certainly, with the ITS-G5, we found it very difficult to identify to find Onboard unit supplier. We felt that at the time the onboard unit market was not very mature.*’

(E1)

Experts mentioned everybody is learning something while they work in this new market.

‘*I think it’s relatively a young industry and young technology. And everyone’s really been learning whilst they’ve been working in C-ITS*’

(E1)

‘*There are only two UK suppliers of the motorized equipment. So, it’s not a competitive market*’

(E2)

This situation leads to two important problems. One of them is to provide a common software and security standard for C-ITS services. Each city experiences new things and tries to learn and understand these systems, and each city develops their individual application. This creates a public barrier:

‘*A lot of people have apps on their phones. Will they want to download another one? And if my app works in Newcastle, then I drive to Leeds or London or Manchester. Do I need to download another app? And, it’s all right me saying I want to manage the city’s roads, but will individuals really engage and use?*’

(E2)

‘*You have to update systems all the time because of security issues and patches. There are things that get older and become out of date and people receive security patches, then it doesn’t work, or everything needs to be patched to the same standard before it starts to work again*’

(E4)

The other problem is legal and political issues because of technical problems. Some participants mentioned that, while the immature technologies make the legal side of C-ITS complicated, these also prevent political awareness.

‘*The legal side of the contracts to do with supplying onboard units was quite complicated and caused some delays for us*’

(E1)

‘*Politicians tend to be a little bit less wanting to take risks in this kind of area*’

(E3)

‘*It’s rare that we have a politician that is super interested in this topic. I mean, I think that the big barrier to C-ITS is does it actually work? Or is this just an interesting technological solution?*’

(E3)

The findings show that technical problems with C-ITS are often caused by these technologies being new and emerging technologies. There are some problems regarding ITS-G5 technologies and in-car technologies. The most important issues for them are insufficient technical support and technical knowledge. The market is so new, and there are not enough knowledgeable suppliers. The other important point about technical knowledge is that these technologies are also new for cities. In addition, the findings show that cities experiencing some problems because of lack of technical support, as well as personal and common technical standards. These lead to some problems, especially security updates, which are necessary to ensure continuity of systems.

#### 3.2.6. Theme 6. Future Deployment and COVID-19

The transportation sector was one of the sectors most affected by COVID-19 worldwide. The data collection took place during the COVID-19 pandemic. Some participants doubted whether public transportation would continue at the same level in the post-coronavirus era. They mentioned that we have always supported public transportation until now, but now we say do not use it. After the COVID-19 period, the transportation trends could change. This situation can change the whole transportation sector and, therefore, C-ITS deployment. Some example quotes are as follows:

‘*So, I’ve mentioned that one of the major customers of the city is a bus operator. Going forward, will they be operating the same level of services that they were operating before COVID and will there be many passengers using their services? So, there’s a question about the viability of some public transport services in particular, and public transport. Road based public transport is one of the key beneficiaries of C-ITS in pretty much every deployment around the country. How COVID continues to impact transport and every area of the economy? There may be changes in lifestyle, more people might work at home, for example, and not use public transport for commuting*’

(E1)

‘*I have no congestion on my network today. So, I don’t need C-ITS*’

(E3)

Participants have mentioned that, in the short term, it is hard to provide technical support and necessary equipment.

‘*The technical people from Siemens haven’t been able to visit to make sure it’s all working. And they weren’t allowed to travel. So that was a barrier*’

(E1)

‘*We’ve not been able to get equipment to the operators to then install on the buses. I was speaking to XXX a couple of days ago. And they were saying that they’ve got problems with procurement, you know, trying to get hold of bits of kit to put on straight. And if it involves a chain of suppliers, or third parties, and everyone’s working at home, and no one’s actually in the warehouse delivering this stuff. So, in the short term, that’s one issue*’

(E4)

In addition to technical problems, evaluation is also an important issue in this period. E4 provided that:

‘*If we’re trying to evaluate C-ITS and say how wonderful it is. And then, unfortunately, you can’t really do evaluation when the traffic’s not the way the traffic usually is. You know, you get erroneous results or whatever*’

(E4)

Participants also stated that C-ITS can have an important role in the post-COVID, world as it combines a wide range of technologies.

‘*There’s a role for C-ITS in mobility as a service in a post COVID world for things like long distance travel, you know, maybe better integration so that you’re not waiting around airports or whatever, where large groups of people could potentially congregate*’

(E4)

‘*It may be that certain C-ITS solutions, particularly ones less focused around cars, might be able to support with social distancing or support with the bus services or something like that*’

(E3)

The European Commission [22] suggests that COVID-19 affected transport and connectivity in Europe significantly. The Commission report shows that there was an approximately 90% reduction in air traffic and 85% reduction in rail and road passenger services in mid-April 2020, when compared to the previous year’s figures. The Commission report provides a roadmap for future transport activities in Europe. It is important to mention that it may take a long time for transportation services to reduce the impact of the coronavirus, and there may be some important changes in transportation movements in the post-COVID-19 period. In this context, experts’ opinions showed that there will be uncertain short-term and long-term effects of COVID-19 to C-ITS deployment.

Short term effects can be divided into two. Participants indicated that, in the short term, the coronavirus has significantly negatively affected the provision of technical services to existing services, and many business partners were seriously affected by this crisis. Additionally, there will be some problems providing enough equipment because industry has been affected significantly. The other important short-term impact is the evaluation of projects. E4 stated that all transport activities changed significantly. Especially, from March to June 2020, and almost all road transport stopped in Europe. The evaluation problems can also be a long-term effect because transport activities may be changed.

The most important long-term impact is transportation behaviour changes. The [22] report showed significant changes in public transport usage, with an almost 85% reduction in April 2020. Participants were concerned that public transport usage trends and, therefore, private car usage numbers, can change. This can affect C-ITS deployments significantly because previous themes showed that business factors are important for deployment, and public service providers are important stakeholders to deploy the services. Additionally, acceptance factors showed that the stakeholders’ acceptance is key. Participants also said that C-ITS services can play an important role in mobility as a service and social distancing, thanks to its innovative technologies in the post-COVID-19 period. The previous themes also showed that one of the most important things for deployment is a good plan. The literature review showed that C-ITS technologies are highly innovative—if a good deployment plan can be provided, the technologies can be used very beneficially in the post-COVID-19 period.

### 3.3. Recommendations and Key Issues to Be Considered for Future Deployment of C-ITS Services

#### 3.3.1. The Additional Needs of Cities during the Implementation and Operation Phases of C-ITS

The findings of this study have identified some important needs, in order to implement and operate C-ITS services effectively. This section presents a list of key issues to be considered for future deployment of C-ITS services.

First of all, the preparation should correspond with the cities’ situation. The following questions should be considered before the effective implementation of C-ITS services:

- How can C-ITS help our city’s mobility challenges?
- Which C-ITS services would be more beneficial for the city?
- How can we effectively and innovatively use the existing infrastructure to deploy C-ITS services?
- How much will C-ITS cost to implement, operate, and maintain?
- Do I have a good business case?

A user-friendly human–machine interface is important, in terms of an effective operation of the C-ITS services [10,23]. When designing the human–machine interfaces of the C-ITS services, the following questions should be considered.

- What information do we need to provide to drivers?
- How much and how often does information need to be provided?
- Will reliable and understandable information be provided?
- Will there be continuity in providing information to drivers?

In addition, the following issues should also be considered.

- What are the expectations of commercial vehicle drivers, and will they be met?
- Are there enough knowledgeable personnel to operate and maintain C-ITS systems?
- Is there good communication between stakeholders?

The above questions provide a summary of the research findings about the objectives.

#### 3.3.2. Major Barriers for a Large-Scale Future C-ITS Deployment, According to Field Experiences of C-ITS’ Experts

This study has identified important barriers to the deployment of C-ITS services. They can be grouped into five categories—financial barriers, organizational barriers, technical barriers, social barriers, and COVID-19 barriers.

Financial Barriers

- C-ITS implementation could be expensive.
- The private sector and policy makers need to see the real benefits of C-ITS to obtain enough funding for C-ITS projects.
- Poor business cases and evaluation prevent suitable funding.
- C-ITS projects are generally so small (there is not necessarily a clear and tangible benefit to the whole of the network, the whole of society, or everybody using the road. Because of that, it is difficult to make an effective cost-benefits analysis. For example, the webTAG in England is not suitable for C-ITS projects).

Organizational Barriers

- A very good standardization infrastructure between cities and countries is necessary.
- It is necessary to improve coordination between cities. Developing a common software for applications, understanding best practices, and learning key faults, thanks to coordination, are important.
- A comprehensive local and private stakeholders’ partnership is necessary.

Technical Barriers

- Many C-ITS technologies are not yet mature enough and continue to evolve.
- Local authorities are not sufficiently experienced with C-ITS.
- The market is so new, and there are not enough knowledgeable suppliers.
- Technical support and maintenance can be difficult, due to partners’ limited knowledge of these new technologies, their inexperience with these technologies, and the lack of standardization.

Social Barriers

- Users may lose interest in these technologies, due to poor design, unreliability, and negative experiences.
- Concerns that C-ITS technologies may distract drivers, especially due to poor human–machine interface.

COVID-19 Barriers

- In the short term, the coronavirus can affect the provision of a technical service to existing services.
- One of the sectors most severely affected by the coronavirus crisis was the transportation sector. This could seriously affect C-ITS business partners financially and in terms of equipment supply.
- In the long run, people’s transport behaviour may change. Less use of public transport and an increase in individual vehicle use can affect C-ITS deployment planning.

## 4. Conclusions

Cooperative intelligent transport systems allow vehicles to communicate with each other and enable cars to communicate with infrastructure, which potentially enhances road safety, optimises traffic management, enhances end-user experiences, improves energy efficiency, and reduces traffic emission and congestion, especially in urban areas [7,10]. To achieve these benefits, a successful and effective deployment of the C-ITS services is imperative. However, limited research has focused on understanding the deployment of C-ITS services, from the perspective of the key stakeholders who have been actively involved in the deployment process. Knowledge regarding the key issues and barriers of a large-scale deployment of the C-ITS services is missing. In light of this, the current study focused on qualitatively examining the C-ITS deployment from the stakeholder perspective. It has advanced a new understanding towards the C-ITS services deployment and identified key issues for future large-scale deployment from six core themes—cost, acceptance, effectiveness, cooperation, technical issues, and future deployment and COVID-19.

The study found that the stakeholders believed that C-ITS services could provide great benefits, in terms of managing traffic in cities, improving transport safety and efficiency, facilitating clean transport systems, and reducing the need for motorised transport, through enhancing the modal shift. The study concluded that, although the potential mobility, social, and environmental benefits of the C-ITS services have been recognised by cities, their potential has not been fully achieved, due to the fact that some of the C-ITS applications are still underdeveloped and incomplete, for example, some of the C-ITS services are not available during busy hours of urban traffic. In addition, a unified and effective method for evaluating the benefits of C-ITS services deployed in cities is needed. This study looked at the benefits, needs, and barriers of C-ITS, with real experience from the key stakeholders. Thus, important issues and barriers to be addressed for future deployment were brought to light (in Section 3.3). Future studies of C-ITS systems could conduct more analysis based on these issues and barriers. The findings of this study provide a new insight into the deployment of C-ITS in cities, which potentially contributes to the preparation of a roadmap for facilitating an effective and successful deployment in the future.

The study yielded useful findings; however, there are still limitations. To begin with, the data collection took place during the COVID-19 pandemic, which limited the recruitment of stakeholders and experts of C-ITS, whilst future research could envision the current findings, using a larger number of stakeholders, for example, 10 to 18. The experts’ roles, tasks, responsibilities, years of experience, and study and research backgrounds within the C-ITS deployment environment should be clearly specified, in order to better understand their requirements and suggestions for the future C-ITS deployment. Additionally, the current study is qualitative in nature, and future research could generalise the findings of this study using a quantitative methodology. The current study identified that user acceptance is important for a successful deployment of C-ITS services, and future research could explore the acceptance and requirements of end users from different social demographic groups [24,25], which potentially contribute to the development and deployment of inclusive C-ITS services. Finally, along with the deployment of C-ITS services, the fast development of automated vehicles potentially deliver new benefits and challenges, in terms of traffic management and pedestrian–vehicle interactions, and future studies could investigate how the C-ITS could work with automated vehicle to facilitate a safe, efficient, and low carbon emission future of mobility [14,26,27].

## Figures and Tables

**Table 1:** Participant background description.

Participant Code	Description
E1	E1 has a significant background knowledge about C-ITS, having attended many EU funded projects. Currently, while carrying out large multinational projects, such as the C-Mobile project, he is also performing much new research at the academic level about C-ITS.
E2	E2 is a transport manager at a local authority. He has great experience in traffic management, C-ITS implementation, traffic management centres, and related technologies.
E3	E3 is also a transport manager at a local authority and manages C-ITS projects in the UK.
E4	E4 has a significant academic background in C-ITS. He is an expert especially in the evaluation of C-ITS projects. He is expert in many technical aspects of C-ITS, including communication systems, related software, and hardware systems.

**Table 2:** Word Frequency Query Result for the first 15 words in NVivo 12.

Word	Length	Count	Weighted Percentage (%)	Similar Words
costs	5	19	002	cost, costs
services	8	20	002	available, help, service, services
operators	9	23	002	controls, engagement, locked, operating, operational, operations, operator, operators, perform, process, work, working, works
technology	10	17	002	engine, engineering, technical, technologies, technology
see	3	21	002	controls, determine, figures, find, learning, looking, project, projects, regard, see, understand, view, visit
need	4	19	002	involved, involvement, necessarily, need, requires, want
city	4	14	001	cities, city
quite	5	16	001	quite, stop, stopped, stopping
lot	3	13	001	lot, much
make	4	23	001	building, cause, form, get, getting, gives, make, makes, making, work, working, works
benefits	8	12	001	benefit, benefits
deployment	10	11	001	deployed, deployment, deployments
key	3	11	001	identify, key
units	5	11	001	unit, units, whole
use	3	11	001	role, use, used, useful, using
ıts	3	11	001	ıts
transport	9	11	001	sending, transport
barriers	8	10	001	barrier, barriers
mobility	8	10	001	mobile, mobility

**Table 3:** Core Themes.

**Main Themes**	**Description**
1. Cost - Implementation Costs - Operation and Maintenance Costs - Whole Life Cost Analysis	Cost is key for C-ITS deployment. A comprehensive price analysis covering all processes is required for the provision and improvement of C-ITS services in cities.
2. Acceptance	Individual vehicle users, commercial vehicle companies, and drivers must accept these services, in order to extend the services.
3. Effectiveness	Cities must be sure of C-ITS benefits.
4. Cooperation - Partnership - Standardization	Good cooperation and a certain standard between partners, cities, and countries are essential for the deployment of C-ITS services.
5. Technical Issues	C-ITS technologies are new, and many technologies still continue to evolve. This situation brings some technical problems.
6. Future Deployment and COVID-19	One of the areas hardest hit by COVID-19 is undoubtedly the transportation sector. This can greatly affect road transport and, therefore, C-ITS services.

## Data Availability

The data that support the findings of the current study are available on request from the corresponding author.
